# POEMS syndrome with cardiovascular lesions as the initial manifestation: a case report and literature review

**DOI:** 10.3389/fcvm.2026.1716667

**Published:** 2026-01-30

**Authors:** Xiuwei Tan, Gaowei Fan, Jiateng Yao, Changwei Lu, Zhaojie Qin

**Affiliations:** Hechi Hospital, The First Affiliated Hospital of Guangxi Medical University, Hechi, Guangxi, China

**Keywords:** cardiovascular lesions, peripheral neuropathy, POEMS syndrome, polycythemia, sclerotic bone lesion

## Abstract

Polyneuropathy, organomegaly, endocrinopathy, monoclonal protein, and skin changes (POEMS) syndrome is frequently mistaken for chronic inflammatory demyelinating polyneuropathy, as its clinical profile is highly variable and neurological symptoms often dominate the initial course. In the present case, the disorder initially presented as cardiovascular dysfunction, which delayed decision-making related to the original diagnosis. As the condition advanced, the patient developed progressive limb weakness, splenomegaly, and abnormalities in lipid metabolism. Whole-body bone scintigraphy revealed osteolytic lesions in association with a plasmacytoma, whereas the serum vascular endothelial growth factor levels were markedly elevated. Based on the cumulative findings, POEMS syndrome was diagnosed. Treatment with interventional chemotherapy combined with adjunctive symptomatic care resulted in a marked reduction in symptoms and promoted functional recovery. This case provides detailed clinical evidence highlighting the critical importance of early recognition and accurate diagnostic evaluation in POEMS syndrome. By aligning our observations with the existing literature, we emphasize strategies that promote timely diagnosis, minimize diagnostic error, and improve therapeutic outcomes.

## Introduction

POEMS syndrome is an uncommon plasma cell disorder, and its name is derived from the acronym representing its characteristic features: Polyneuropathy (P), Organomegaly (O), Endocrinopathy (E), Monoclonal protein (M), and Skin changes (S) (1). Beyond these classic features, the clinical picture may be broadened to include sclerotic bone lesions, Castleman disease, papilledema, peripheral or generalized edema, ascites, elevated platelet counts, polycythemia, thrombotic complications, and multiorgan involvement affecting the kidneys, lungs, and heart (2). From a pathophysiological perspective, two diagnostic hallmarks are particularly notable: markedly elevated vascular endothelial growth factor (VEGF) levels, reflecting a cytokine-mediated inflammatory cascade, and the presence of a monoclonal lambda (*λ*) immunoglobulin (3, 4). When compared with chronic inflammatory demyelinating polyneuropathy (CIDP), POEMS syndrome demonstrates more pronounced axonal injury in the peripheral nerves, with the lower limbs more severely affected than the upper limbs (5). Neuropathy typically begins with sensory disturbances such as paresthesia or hyperesthesia and gradually progresses to profound motor deficits, which include distal muscle weakness (e.g., foot drop), muscle atrophy, areflexia, and gait abnormalities (6, 7). This relentless neurological decline is a key determinant of both morbidity and mortality in the affected individuals (8). Because this disorder is rare, with a prevalence estimated at only 0.3 per 100,000 individuals (9) and presents with heterogeneous features, misdiagnosis is common. It is frequently mistaken for CIDP, which often results in diagnostic delays and missed opportunities for timely intervention (10). To minimize these risks, accurate recognition requires a meticulous diagnostic work-up that integrates detailed medical history, comprehensive clinical examination, and a collaborative, multidisciplinary assessment (11). In this report, we have described a case of POEMS syndrome that first manifested with cardiovascular abnormalities to provide a focused clinical analysis.

## Case presentation

A 34-year-old man presented to our hospital with a 2-month history of persistent lower back pain, progressively accompanied by numbness and weakness in both lower limbs; 10 days prior, he had been evaluated at a local hospital, where he was diagnosed with “lumbar disc herniation,” for which he received inpatient care. Owing to inadequate symptom relief following the standard therapy, he was referred to our institution for further assessment and management.

The patient's medical history was notable for a prior admission to the Cardiology Department of our hospital from January 29 to February 2, 2024, prompted by chest tightness lasting for >10 days. At that time point, the working diagnoses included “suspected coronary artery disease under observation” and hyperlipidemia. Physical examination revealed no hepatosplenomegaly, absence of lower limb edema, and intact neurological function. He was discharged with a prescription for aspirin enteric-coated tablets and atorvastatin calcium tablets.

Upon current evaluation, physical examination revealed multiple enlarged, firm lymph nodes palpable in the bilateral submandibular and inguinal regions. The lumbar spine maintained normal physiological alignment; however, sensory testing demonstrated a diminished perception of pain and light touch in both lower limbs, accompanied by mild tenderness over the L5–S1 paravertebral area. Neurological assessment revealed the complete absence of deep tendon reflexes in the brachioradialis, biceps, triceps, patellar, and Achilles tendons bilaterally. Manual muscle testing revealed a strength of 3/5 in the tibialis anterior, extensor hallucis longus, and gastrocnemius muscles bilaterally, and 4/5 in the triceps brachii. No pathological reflexes were elicited. Electromyography indicated impaired conduction across the peripheral nerves in the upper and lower limbs, which is consistent with moderate generalized peripheral neuropathy.

## Auxiliary examinations

### Laboratory examinations

At the time of readmission, following an earlier misdiagnosis of “lumbar disc herniation” for bilateral lower limb numbness, the patient underwent repeat evaluation of cardiovascular parameters on November 11, 2024. The findings were as follows: lipid profile: total cholesterol = 2.62 mmol/L, high-density lipoprotein cholesterol = 0.61 mmol/L, and low-density lipoprotein cholesterol = 1.38 mmol/L; inflammatory markers: C-reactive protein = 9.8 mg/L and interleukin (IL)-6 = 9.50 pg/mL; coagulation and hematology: fibrinogen = 4.41 g/L and erythrocyte sedimentation rate = 21 mm/h; complete blood count: platelets = 354 × 10^9^/L, mean corpuscular hemoglobin = 26.6 pg, monocytes count = 0.67 × 10^9^/L, and red blood cell count = 5.91 × 10^12^/L; and VEGF = 1,262.34 pg/mL. With lumbar disc herniation effectively excluded, POEMS syndrome became the primary diagnostic consideration. Additional laboratory tests performed at this stage, including hematologic indices, coagulation profile, myocardial enzyme assays, and serum electrolytes, are presented in Table 1.

**Table 1:** Laboratory test outcomes during November 10–20, 2024.

Test category	Parameter	Result	Test category	Parameter	Result
Coagulation panel	Plasma prothrombin time	10.2 S	Electrolyte panel	Potassium level	4.6 mol/L
	Activated partial thromboplastin time	25.0 S		Sodium level	139.3 mol/L
	Thrombin time	18.5 S		Chloride level	101.6 mol/L
	Plasma fibrinogen	2.27 g/L		Calcium level	2.29 mol/L
Complete blood count (differential)	White blood cell count	**10.92 × 10^9^/L**		Lymphocyte count	**5.13 × 10^9^/L**
	Neutrophil percentage	42%		Monocyte count	**0.85 × 10^9^/L**
	Lymphocyte percentage	47%		Eosinophil count	**0．18 × 10^9^/L**
	Monocyte percentage	7.8%		Basophil count	**0.17 × 10^9^/L**
	Eosinophil percentage	1.60%		Red blood cell count	5.65 × 10^12^/L
	Basophil percentage	**1.60%**		Hemoglobin level	153.00 g/L
	Neutrophil count	4.59 × 10^9^/L			
Cardiac enzyme panel	Serum creatine kinase level	71 U/L	Renal function panel	Urea level	5.99 uoml/L
	Serum creatine kinase-MB Isoenzyme activity	19 U/L		Creatinine level	73 uoml/L
	*α*-Hydroxybutyrate Dehydrogenase level	102 U/L		Urea-to-creatinine ratio	0.08
	Lactate dehydrogenase level	163 U/L		Serum bicarbonate (HCO₃⁻) level	29.1 uoml/L
	Serum lactate dehydrogenase isoenzyme level	31 U/L		Uric acid level	**544 uoml/L**
	Serum *β*2-microglobulin level	**2.3 mg/L**			

Boldface type indicates values out of range.

### Imaging examinations

**Figure 1:**
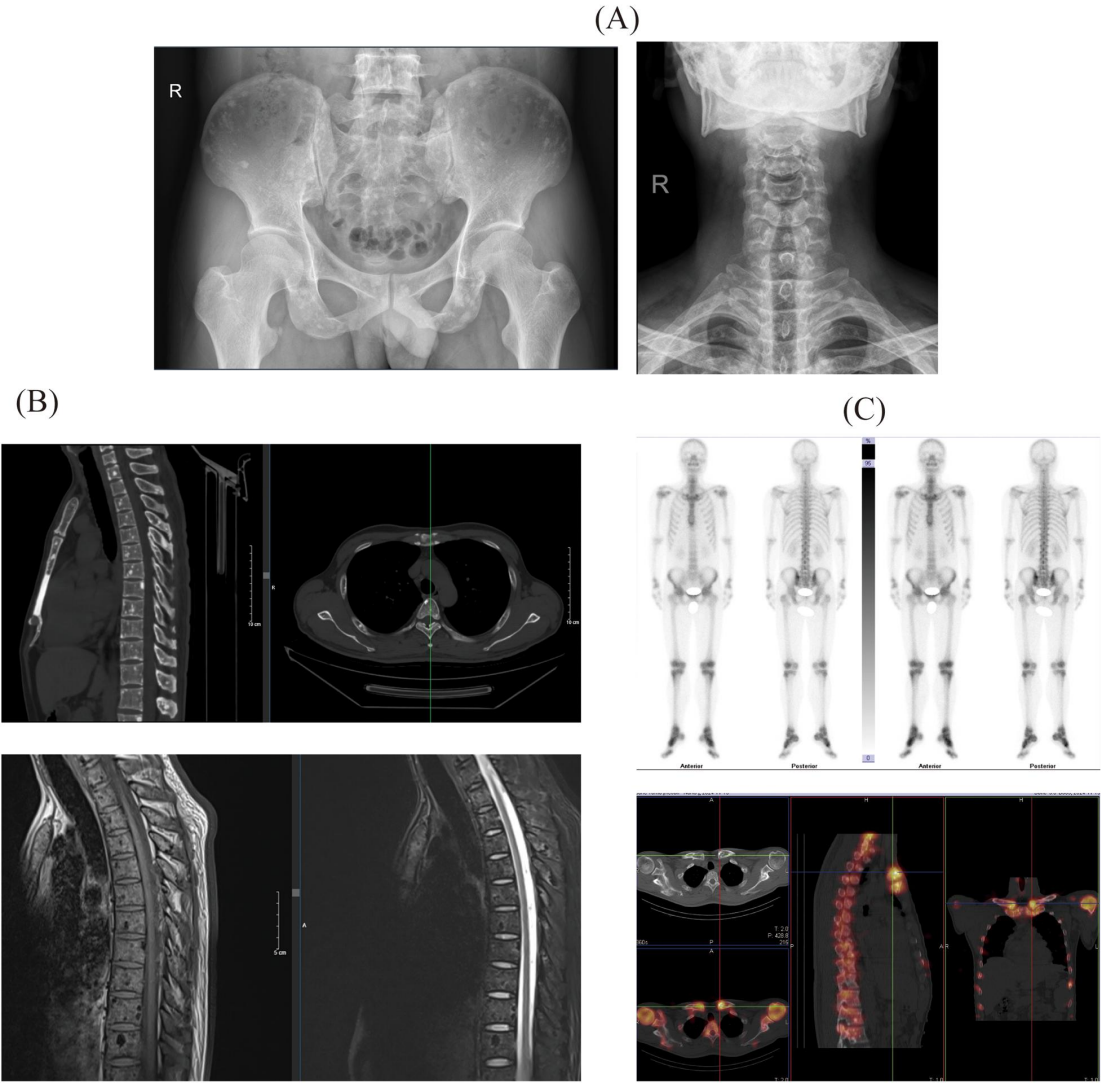
The results of the patient's whole-body imaging examination include: **(A)** x-ray images of the pelvis and cervical spine; **(B)** computed tomography (CT) and magnetic resonance imaging (MRI) results of the spine; **(C)** single-photon emission computed tomography (SPECT) results of the patient.

Radiographic Imaging: Plain x-ray demonstrated diffusely distributed, small nodular areas of slightly increased density involving the vertebral bodies, their appendages, pelvic bones, and ribs, with indistinct margins and no associated surrounding soft tissue masses. Marginal osteophytes were observed along the cervical vertebrae, and multiple nodules of varying sizes with low signal intensity were detected across all sequences in the vertebrae and their appendages, exhibiting relatively well-defined boundaries (Figure 1A).

Computed Tomography (CT): CT imaging identified diffusely scattered punctate and nodular hyperdense lesions with clear margins affecting the spine, its appendages, and the pelvic bones. Magnetic Resonance Imaging (MRI): MRI revealed multiple nodules of varying size, exhibiting low signal intensity across all sequences, which were noted within the vertebral bodies and appendages, with no enhancement following contrast administration (Figure 1B).

Single-Photon Emission Computed Tomography (SPECT): SPECT imaging demonstrated diffusely distributed punctate, nodular, and larger patchy hyperdense areas within the spine and its appendages, sternum, both shoulder joints, and bilateral ribs, clavicles, and humeri (Figure 1C).

**Figure 2:**
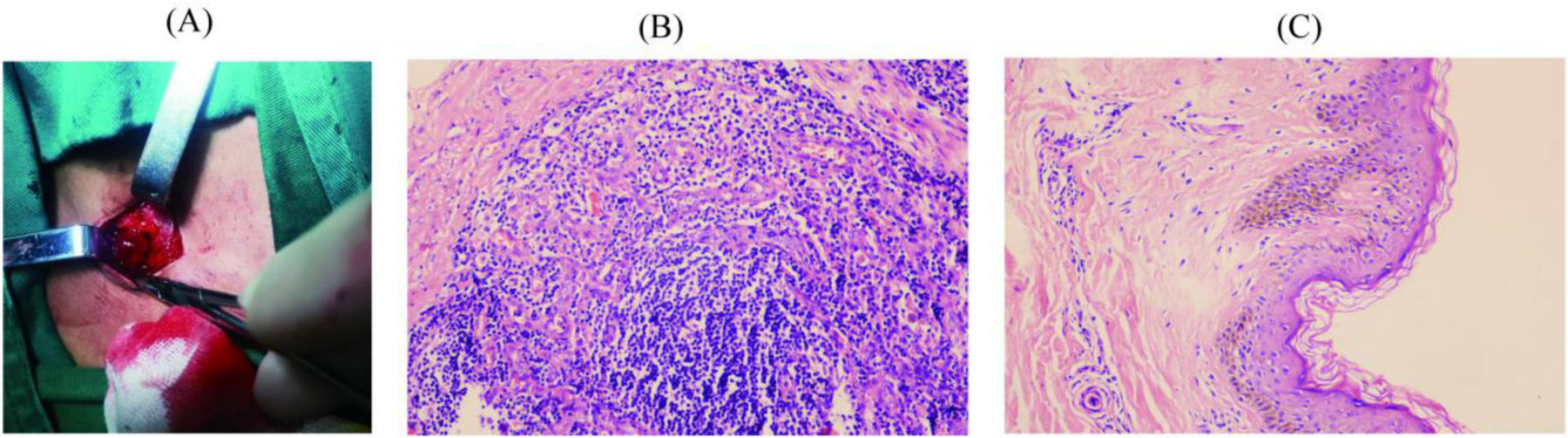
Clinical samples and hematoxylin-eosin staining results of the patient. **(A)** Inguinal lymph node sample; **(B)** Pathological findings of the left inguinal lymph node; **(C)** Pathological findings of the left inguinal skin.

Pathology and Immunohistochemistry: Histopathological analysis of the left inguinal lymph node, performed on November 14, 2024, revealed chronic inflammation with reactive hyperplasia (Figure 2B). The examination of the left inguinal skin demonstrated chronic inflammatory changes, thinning of the squamous epithelium, and basal cell layer pigmentation (Figure 2C).

**Figure 3:**
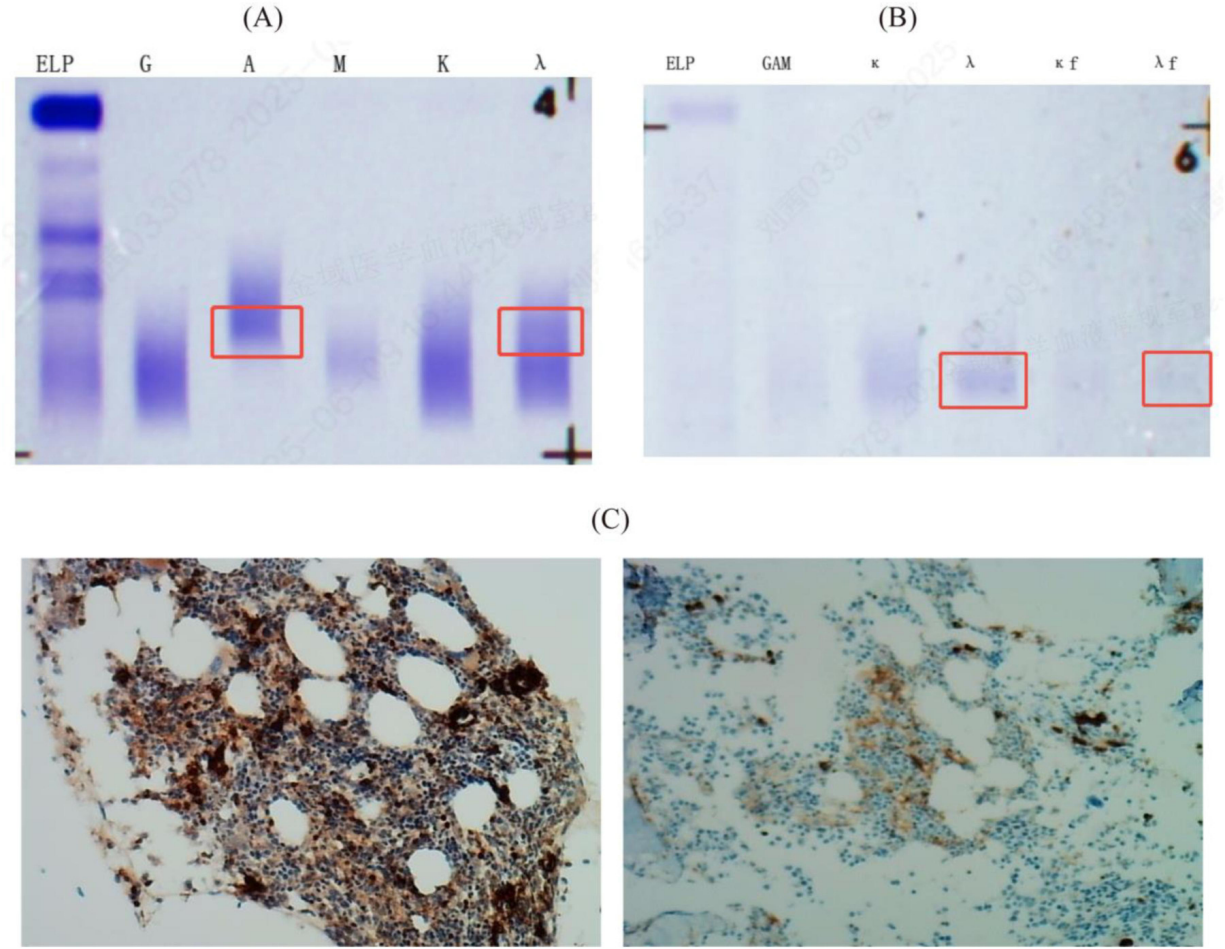
Results of serum immunofixation electrophoresis (IFE), urine bence-jones protein electrophoresis (BJP) and immunohistochemistry in the patient. **(A)** Serum IFE showed a monoclonal immunoglobulin type of IgA-*λ* in the IgA, IgG, IgM, *κ*, and *λ* lanes; **(B)** Urine BJP was positive, with the type being *λ* free light chain; BJP: Bence-Jones protein; ELP: Immunofixation electrophoresis; G: IgG; A: IgA; M: IgM; D: IgD; E: IgE; *κ*: *κ* chain; *λ*: *λ* chain; *κf*: Free *κ* chain; *λf*: Free *λ* chain. **(C)** CD38 scattered and small clusters (2%, strong +), Kappa scattered few (+), Lambda scattered and small clusters (+), CD19 scattered and 2 small nodules (+), Cyclin-D1 (−), MUM1 scattered and small clusters (+), BCMA (2%, moderate +), GPRC5D (1%, moderate +), P53 (5% +), BCL-2 scattered and 2 small nodules (+), Ki-67 (40% +).

Laboratory and Diagnostic Investigations: Serum immunofixation electrophoresis identified a monoclonal immunoglobulin of the IgA-*λ* subtype (Figure 3A). Urinary Bence–Jones protein analysis revealed precipitation bands corresponding to the *λ*-light chain and free *λ*-light-chain regions, confirming the presence of Bence–Jones protein of the *λ*-free light-chain type (Figure 3B). Immunohistochemical assessment revealed a mild increase in the plasma cells (approximately 2%), which raised suspicion for plasmacytoma (Figure 3C).

Endocrine evaluation showed a serum prolactin level of 16.36 ng/mL and a testosterone level of 97.06 ng/dL. Electromyographic studies demonstrated abnormal nerve conduction in the upper and lower limbs, with neurogenic changes indicative of multiple peripheral nerve injuries. These findings indicated a combination of demyelination and axonal degeneration of moderate severity, consistent with the 2023 mandatory major diagnostic criteria for POEMS syndrome (12).

**Figure 4:**
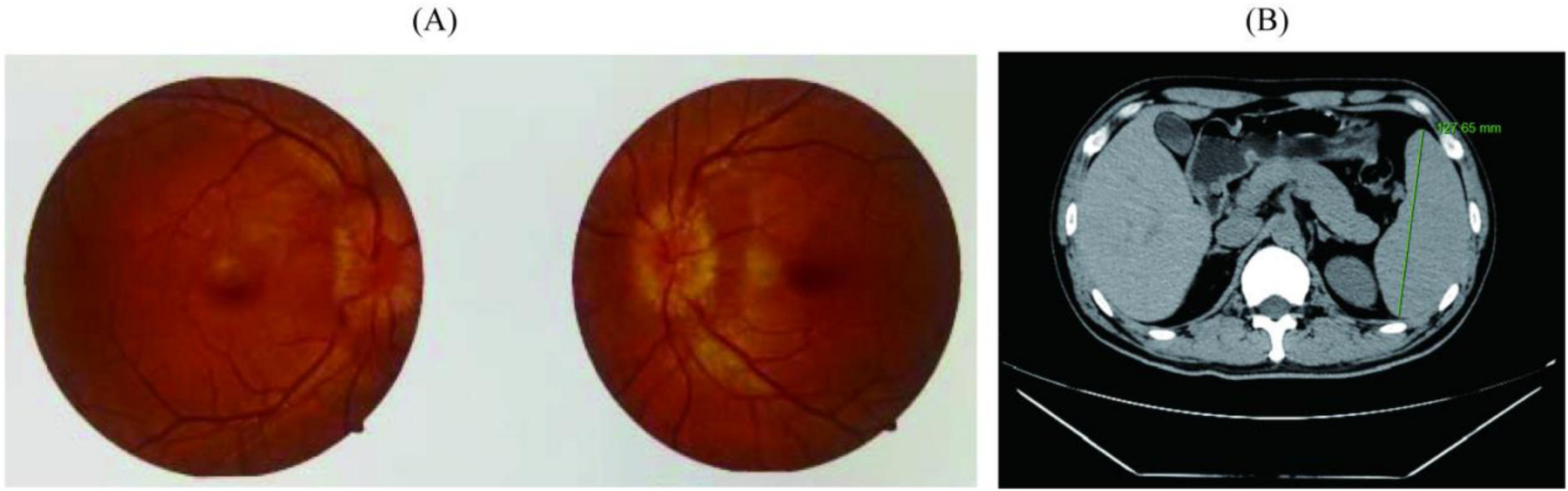
Examination results of patient-related complications. **(A)** Results of fundus optic nerve examination; **(B)** Results of spleen computed tomography scan.

The imaging results corroborated these findings, confirming osteosclerotic lesions and markedly elevated serum VEGF levels. Fundoscopic examination revealed intact macular regions bilaterally, accompanied by optic disc edema (Figure 4A). CT of the abdomen revealed splenomegaly, with a maximal longitudinal dimension of 127.65 mm and a thickness of approximately 5.0 cm at the splenic hilum (Figure 4B).

Following a multidisciplinary consultation and comprehensive integration of clinical, laboratory, and imaging findings while systematically excluding alternative diagnoses, a definitive diagnosis of POEMS syndrome was established. Renal function parameters pre- and post-treatment are summarized in Table 2.

**Table 2:** Renal function parameters of the study patients before and after treatment.

Time of assessment	Uric acid level	β2-microglobulin level
After Treatment	243 uoml/L	**4.8 mg/L**
Before Treatment	**496 uoml/L**	2.3 mg/L

Boldface type indicates values out of range.

## Treatment and outcome

Following confirmation of the POEMS syndrome, the patient was initiated on a therapeutic regimen on November 20, 2024, comprising lenalidomide (25 mg orally on days 1–21) and dexamethasone (20 mg intravenously on days 1, 8, 15, and 22 of each 28-day cycle). Adjunctive supportive measures included aspirin enteric-coated tablets to inhibit platelet aggregation, atorvastatin calcium tablets for plaque stabilization, mecobalamin to support peripheral nerve repair, rabeprazole enteric-coated tablets for acid suppression and gastric mucosal protection, and sucralfate suspension gel to reinforce gastric mucosal protection. Follow-up neurological assessments conducted in March and June 2025 revealed substantial clinical improvement, as evidenced by increased muscle strength in both lower limbs, enhanced gait stability, and a notable reduction in fall frequency.

## Discussion

POEMS syndrome is a rare and complex plasma cell disorder that is frequently misdiagnosed in medically underserved regions owing to its broad spectrum of clinical manifestations, often leading to delayed treatment and progressive morbidity (13). Management primarily focuses on two objectives: eradication of the underlying clonal plasma cell population and provision of supportive care to address the syndrome's diverse systemic effects (14).

In our patient, the initial presentation consisted of chest tightness accompanied by a paroxysmal “obstructive” sensation beneath the lower sternum. Coronary artery disease was initially suspected, and symptomatic treatment with Compound Danshen tablets, fluvastatin, and atorvastatin calcium tablets provided only transient relief, with symptoms recurring intermittently. Notably, POEMS syndrome cases that initially present with cardiac involvement tend to follow a more aggressive clinical course (15, 16), potentially progressing to cardiomyopathy, pericarditis, pericardial effusion, or heart failure. Among these complications, heart failure accounts for up to one-third of disease-related mortality (17, 18). Vascular endothelial growth factor (VEGF), a pivotal cytokine in angiogenesis, increases vascular permeability, promotes neovascularization, and contributes to osteoblast activation (19). Elevated VEGF levels play a central role in the development of the characteristic clinical manifestations of POEMS syndrome (2). Therefore, in Latin America, measurement of VEGF is a key component of the diagnostic criteria for POEMS syndrome (20). The majority of patients with POEMS syndrome exhibit clonal proliferation of *λ*-type plasma cells, often with restricted VJ region usage in the bone marrow, reflecting the monoclonal origin of the disease (21). Certain *λ* light chains may engage in molecular mimicry with receptors involved in VEGF secretion, thereby directly promoting VEGF oversecretion (22). Therefore, the combination of unexplained cardiac abnormalities and elevated serum VEGF levels should prompt heightened clinical suspicion for POEMS syndrome.

Skeletal lesions in POEMS syndrome typically progress slowly through sclerotic or reparative processes (23). Therapeutic interventions directed at the underlying plasma cell clone can promote re-ossification of osteolytic lesions and stabilization of sclerotic bone changes, potentially reducing compressive effects on adjacent neural structures (24). The neurological manifestations in this case were prototypical and provided critical diagnostic clues. The patient presented with bilateral lower limb numbness and weakness, and electromyography demonstrated a mixed pattern of moderate demyelination and axonal injury, consistent with the characteristic peripheral neuropathy of POEMS syndrome. It has been established that dysregulated VEGF levels are closely associated with systemic skeletal abnormalities and vascular pathology, including coronary atherosclerosis. Effective reduction of VEGF can simultaneously ameliorate multiple clinical features, such as papilledema and cardiac dysfunction, while normalizing bone metabolism (25, 26). Conversely, VEGF also serves as a sensitive biomarker of the therapeutic response. In clinical studies of POEMS syndrome, extravascular volume overload has been observed in at least one-third of patients, most commonly manifesting as peripheral edema or serous cavity effusions, including ascites or pleural effusion (27). However, their secondary impact on peripheral nerves, whether through mechanical compression or VEGF-induced endoneurial edema, may be reversible with appropriate therapy. Furthermore, involvement of the motor central nervous system may be more common than previously recognized (28). In studies by Maroun et al., systemic therapy not only achieved hematologic remission but also produced a rapid decline in serum VEGF levels (29, 30). This biochemical improvement was subsequently associated with measurable recovery of neurological function, often accompanied by stabilization or partial restoration of neurophysiological parameters (31). The underlying mechanism likely involves a reduction in VEGF-mediated vascular permeability, resulting in decreased endoneurial edema and the restoration of a microenvironment conducive to nerve repair (32). In cases where clinical evaluation reveals suggestive features, such as cutaneous changes or pedal edema, assessment of VEGF levels should be strongly prioritized (33). Concurrently, vigilant monitoring of organ involvement is essential, as pathological changes may arise in the spleen, liver, or lymph nodes, which are components of the ancillary diagnostic criteria, as the disease progresses (34).

Given the broad and variable presentation of POEMS syndrome, patients who initially present with lower limb weakness are at high risk of being misdiagnosed with other neurological disorders, particularly chronic inflammatory demyelinating polyradiculoneuropathy (CIDP) (35). Therefore, key strategies to minimize early stage misdiagnosis include maintaining a high index of suspicion for POEMS in patients with non-specific polyneuropathy syndromes; incorporating serum VEGF measurement and M-protein screening into the routine workup for neuropathy workup; and recognizing POEMS as a systemic disorder that necessitates multidisciplinary collaboration spanning neurology, hematology, endocrinology, and radiology, for diagnosis and effective management.

Early involvement of a multidisciplinary team, together with improved clinician expertise in distinguishing POEMS syndrome from other disorders and a deeper understanding of its pathophysiology, can substantially reduce the risk of delayed or missed diagnosis. Additionally, enhancing patient education and increasing disease awareness can facilitate earlier recognition and timely intervention. Collectively, these strategies may transform the management of POEMS syndrome from a historically challenging condition into one that is increasingly identifiable, controllable, and responsive to precision therapies.

## Data Availability

The original contributions presented in the study are included in the article/Supplementary Material, further inquiries can be directed to the corresponding author.
